# Pediatric lupus nephritis

**DOI:** 10.1590/2175-8239-JBN-2018-0097

**Published:** 2018-11-14

**Authors:** Sergio Veloso Brant Pinheiro, Raphael Figuiredo Dias, Rafaela Cabral Gonçalves Fabiano, Stanley de Almeida Araujo, Ana Cristina Simões e Silva

**Affiliations:** 1 Universidade Federal de Minas Gerais Hospital das Clínicas Unidade de Nefrologia Pediátrica Belo HorizonteMG Brasil Universidade Federal de Minas Gerais, Hospital das Clínicas, Unidade de Nefrologia Pediátrica, Belo Horizonte, MG, Brasil.

**Keywords:** Lupus Nephritis, Pediatrics, Autoimmunity, Antibodies, Antinuclear

## Abstract

Involvement of the kidneys by lupus nephritis (LN) is one of the most severe clinical manifestations seen in individuals with systemic lupus erythematosus (SLE). LN is more frequent and severe in pediatric patients and has been associated with higher morbidity and mortality rates. This narrative review aimed to describe the general aspects of LN and its particularities when affecting children and adolescents, while focusing on the disease's etiopathogenesis, clinical manifestations, renal tissue alterations, and treatment options.

## Introduction

Systemic lupus erythematosus (SLE) is a chronic inflammatory condition that affects numerous organs such as the skin, joints, lungs, heart, kidneys, and nervous system.(1) Its etiology is multifactorial and includes genetic and environmental factors. The involved pathophysiological mechanisms include decreased immune tolerance, production of antibodies, deposition of immune complexes on target tissues, and activation of the complement system.(2-4)

Involvement of the kidneys by lupus nephritis (LN) is one of the most severe clinical manifestations observed in individuals with systemic lupus erythematosus (SLE). LN is more frequent and severe in pediatric patients and has been associated with higher morbidity and mortality rates.(5,6) This review aimed to describe the general and particular features of LN in children and adolescents and to shed light on the disease's etiopathogenesis, clinical manifestations, histopathology, and treatment.

## Epidemiology

SLE preferentially affects non-Caucasian young women.(7,8) Patients aged 18 years or younger account for up to 20% of the cases.(9) The prevalence of SLE in children and adolescents (juvenile SLE) varies as a function of the ethnicity and age range of the individuals enrolled in different studies.(9) Juvenile SLE is a rare disease, with an incidence of 0.3-0.9/100,000 children per year and a prevalence of 3.3-8.8/100,000 children.(10)

Neonatal SLE is a rare condition that equally affects individuals of both sexes. It is usually associated with maternal SLE and other autoimmune diseases.(11,12) Multicenter studies performed in Brazil and the USA suggested that SLE in infants is usually associated with complement deficiencies.(13,14) Female children and adolescents develop SLE more commonly, possibly due to the hormonal changes of puberty.(15) The predominance of SLE in female pediatric patients increases gradually with age to the values observed in adults.(16-19)

Although similar to the manifestations observed in adults with SLE, the clinical events present in juvenile SLE are usually more severe and involve multiple organs.(1,5,6,20,21) Renal involvement occurs in 50-75% of pediatric patients with SLE and more than 90% develop LN within two years of diagnosis.(1) Individuals aged 10-13 years are preferentially involved and present an incidence of 0,72/100,000 per year.(1,20) The risk of patients with juvenile LN developing LN is higher among Asians, African Americans, and Hispanics.(21)

The 5-year renal survival of children with LN has improved markedly in recent decades, and currently ranges from 77% to 93%.(21) However, when compared to healthy children, the mortality rate seen in pediatric individuals with LN is 19 times greater.(21) The prognosis of children with LN and end-stage renal disease is particularly somber. Mortality rates within the first five years of renal replacement therapy may reach 22%, mainly on account of cardiopulmonary complications.(21)

## Etiopathogenesis

The pathogenesis of SLE involves a complex interaction between genetic susceptibility and environmental factors, which result primarily in loss of immune tolerance and onset of chronic autoimmunity.(22-25 )Genetic susceptibility stems from genetic mutations that may predispose patients to developing SLE.(22-25) Environmental factors induce epigenetic alterations - variations in gene expression caused by DNA methylation and histone modification and/or non-coding RNA - that may trigger the onset of SLE in genetically predisposed individuals. Epigenetic changes may be caused by factors such as viral infection, sun exposure, hormonal alterations, nutrition, physical and mental stress, and medication.(22-25)

Loss of immune tolerance is the initial trigger for SLE.(22-25) Immune tolerance is not lost under normal conditions, since nuclear self-antigens - subsequently to neutrophil apoptosis (NETosis) - rarely persist for long enough to be processed by antigen-presenting cells.(22,25) The clearance of dead cells and genetic material is impaired in SLE on account of apoptosis and NETosis defects, which expose self-antigens to the immune system.(22,25) Some genetic defects of the complement system may introduce flaws in opsonization and thus impair the clearance of self-antigens.(22) Nuclear self-antigen internalization and recognition by toll-like receptors (TLR 2 and 9 in particular) promotes the conversion of dendritic cells into antigen-presenting cells, and consequently the activation of autoreactive T cells.(22-25) By their turn, autoreactive T cells amplify the immune response by increasing the production of T and B cells in the bone marrow and lymphoid organs.(22-25) Active B cells may differentiate into plasma B cells or memory B cells.(22-25) Active B cells continuously exposed to nuclear self-antigens produce large quantities of autoantibodies, which then react with nuclear self-antigens to form circulating immune complexes (CIC).(22-25) CIC are not adequately cleared and deposit in various tissues.(22-25) A few physiological phenomena protect self-DNA against identification by the immune system.(26) Impaired clearance of dead cells and genetic material has been associated with loss of discrimination between self-genetic and viral material by the immune system.(26)

**Figure 1:**
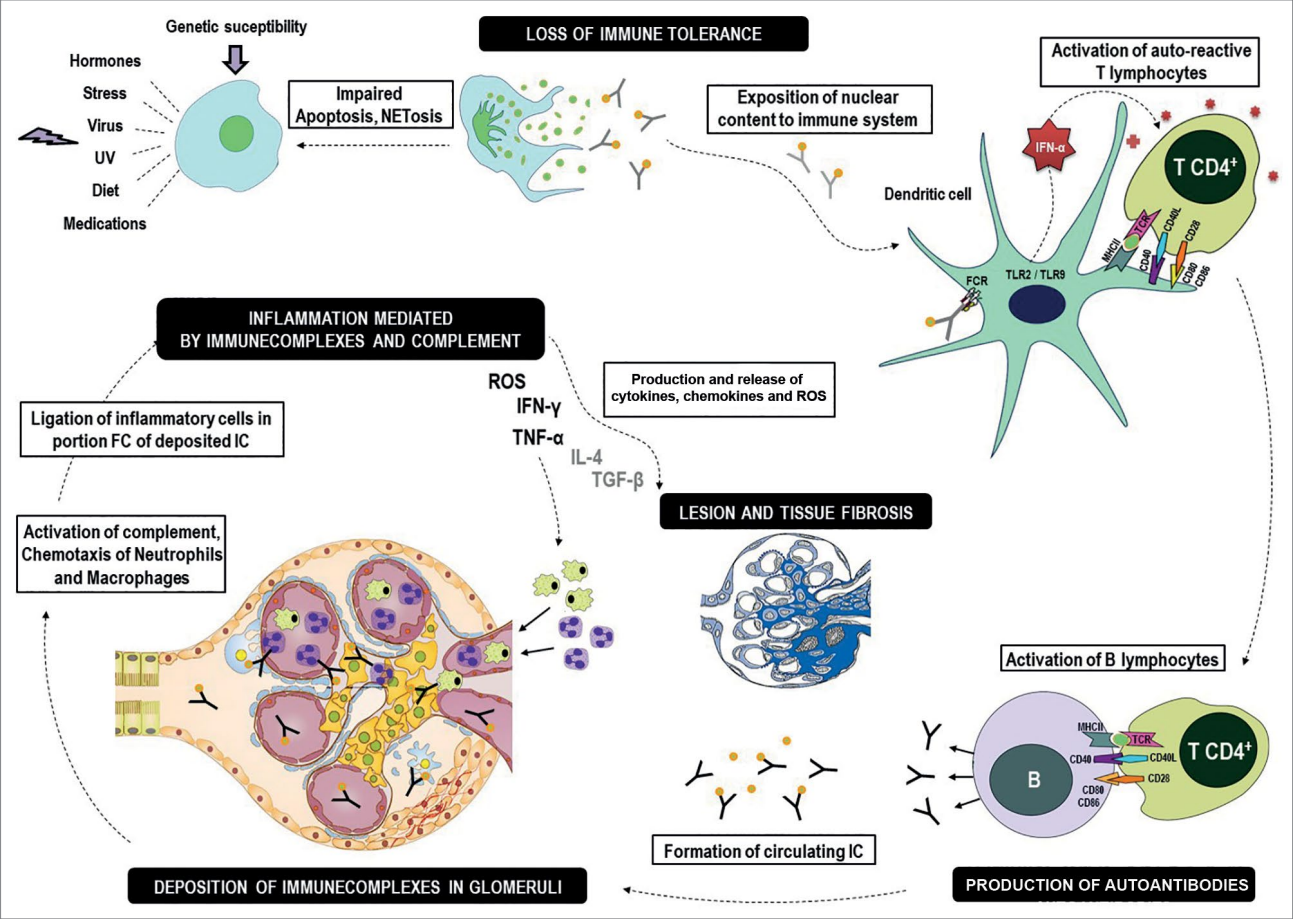
Schematic representation of the pathogenesis of lupus nephritis.

Renal involvement in SLE derives from the deposition of CIC in renal tissue or from the formation of IC in situ (Figure 1)*.*(23-25) The deposition of IC in renal tissue activates the classical complement, macrophage, and neutrophil pathways from the binding of phagocyte surface Fc receptors and immunoglobulin complexes.(23,25) Complement system protein C1q binds to the Fc region of IgG (IgG1 and IgG3 in particular) or IgM present in IC deposits to promote neutrophil activation.(25) The activation of the classical complement pathway leads to the formation of chemoattractant complement system proteins (C3a and C5a), which also induce neutrophil recruitment.(23-25) Local neutrophil activation and recruitment trigger the release of reactive oxygen species (ROS), the production of proinflammatory cytokines, and the amplification of immune and inflammatory response in renal tissues.(23-25) Proinflammatory and profibrotic cytokines [mainly interleukin-4 (IL-4), transforming growth factor-beta (TGF-beta), tumor necrosis factor (TNF), and interferon gamma (IFN-gamma)] induce different grades of podocyte injury, proliferation of mesangial, endothelial, and parietal epithelial cells, increased extracellular matrix synthesis and deposition, and renal impairment.(23-25)

## Clinical manifestations

The glomerulus is the most severely affected structure in the nephrons of individuals with LN.(21) Altered ultrafiltration membrane permeability is a common finding - often associated with proteinuria of varying degrees and local inflammation - linked to glomerular hematuria and decreased glomerular filtration.(21) Glomerular injuries may be focal or diffuse.(21) Therefore, the presentation and clinical development of LN in pediatric patients vary considerably - from benign, slow-progressing cases to rapidly progressing disease.(21) Patients may present with asymptomatic hematuria, mild proteinuria, nephrotic syndrome, acute nephrotic syndrome, rapidly progressive glomerulonephritis, acute or chronic kidney injury.(1,2,5,21,27) In some cases the interstitium and renal tubules may be compromised, thus impairing the mechanisms of urine concentration and electrolyte reabsorption.(2,5,21,27) Despite the large number of clinical manifestations, the signs and symptoms of LN do not always reflect the severity of the disease. Additionally, clinical findings do not predict the clinical development or the prognosis of patients with the disease. Therefore, kidney biopsy becomes an essential measure at assessing tissue involvement, categorizing LN, and choosing the course of therapy.(5,21)

## Complementary workup

### Diagnosis

Early detection of LN is of the essence, since renal involvement may decrease the 10-year survival by 88%.(28) According to the guidelines established by the Systemic Lupus International Collaborating Clinics (SLICC) in 2012, LN may occur in patients diagnosed with SLE or LN alone.(29) The diagnosis of SLE requires patients to present at least four of the criteria defined by the SLICC, including one clinical and one immunological not necessarily occurring simultaneously (Table 1).(29) Renal involvement in patients with SLE is defined by the following: 24-hour urinary protein ≥ 500 mg (or urine protein to creatinine ratio ≥ 0.5) OR red blood cell casts in urine. A possibly ideal additional criterion is renal biopsy showing immune-complex-mediated nephritis with complement deposition associated with varying degrees of cell injury.(30) Renal biopsy must be ordered whenever LN is suspected.(30) In order to be diagnosed with LN alone, patients must have renal biopsy findings consistent with LN along with high levels of antinuclear antibodies (ANA) and/or increased circulating levels of anti-double stranded DNA (anti-dsDNA) antibodies.(29)

**Table 1:** Clinical and immunologic criteria used in the classification of the Systemic Lupus International Collaborating Clinics (SLICC)

Clinical criteria
1. Acute cutaneous lupus, including:
Lupus malar rash (do not count if malar discoid)
Bullous lupus
Toxic epidermal necrolysis variant of SLE
Maculopapular lupus rash
Photosensitive lupus rash
*In the absence of dermatomyositis*
OR subacute cutaneous lupus (nonindurated psoriasiform and/or annular polycyclic lesions that resolve without scarring, although occasionally with post-inflammatory dyspigmentation or telangiectasias)
2. Chronic cutaneous lupus, including:
Classical discoid rash
Localized (above the neck)
Generalized (above and below the neck)
Hypertrophic (verrucous) lupus
Lupus panniculitis (Profundis)
Mucosal lupus
Lupus erythematosus tumidus
Chilblains lupus
Discoid lupus/lichen planus overlap
3. Oral ulcers
Palate
Buccal
Tongue
OR nasal ulcers
*In the absence of other causes such as vasculitis, Behçet's disease, infection (herpesvirus), inflammatory bowel disease, reactive arthritis, and acidic foods*
4. Non-scarring alopecia (diffuse thinning or hair fragility with visible broken hairs)
*In the absence of other causes such as alopecia areata, drugs, iron deficiency, and androgenic alopecia*.
5. Synovitis involving two or more joints, characterized by swelling or effusion
OR tenderness in two or more joints and at least 30 minutes of morning stiffness
6. Serositis
Typical pleurisy for more than one day
OR pleural effusions
OR pleural rub
Typical pericardial pain (pain with recumbency improved by sitting forward) for more than one day
OR pericardial effusion
OR pericardial rub
OR pericarditis by electrocardiography
*In the absence of other causes such as infection, uremia, and Dressler’s pericarditis*
7. Renal
Urine protein-to-creatinine ratio (or 24-hour urine protein) equal to or greater than 500 mg protein/24 hours OU red blood cell casts
8. Neurologic
Seizures
Psychosis
Mononeuritis multiplex
*In the absence of other known causes such as primary vasculitis*
Myelitis
Peripheral or cranial neuropathy
*In the absence of other known causes such as primary vasculitis, infection, and diabetes mellitus*
Acute confusional state
*In the absence of other causes, including toxic/metabolic, uremia, drugs*
9. Hemolytic anemia
10. Leukopenia (< 4000/mm^3^ at least once)
*In the absence of other known causes such as Felty’s syndrome, drugs, and portal hypertension*
OR lymphopenia (< 1000/mm^3^ at least once)
In the absence of other known causes such as corticosteroids, drugs, and infection
11. Thrombocytopenia (< 100,000/mm^3^ at least once)
*In the absence of other known causes such as drugs, portal hypertension, thrombotic thrombocytopenic purpura*
**Immunologic criteria**
1. ANA level above laboratory reference range
2. Anti-dsDNA antibody level above laboratory reference range (or 2-fold the reference range if tested by ELISA)
3. Anti-Sm: the presence of antibody to Sm nuclear antigen
4. Antiphospholipid antibody positivity, as determined by:
Positive test for lupus anticoagulant
False-positive test result for rapid plasma reagin
Moderate titer anticardiolipin level (IgA, IgG, or IgM)
Positive test result for anti-2-glycoprotein I (IgA, IgG, or IgM)
5. Low complement
Low C3
Low C4
Low CH50
6. Direct Coombs’ test in the absence of hemolytic anemia

Source: Petri M et al., 2012.^29^

Notes: The criteria are cumulative and do not have to be present simultaneously.

Anti-dsDNA: anti-double stranded DNA; ELISA: enzyme-linked immunosorbent assay; ANA: antinuclear antibodies.

Patients with SLE may present with numerous renal disorders not linked to LN, such as thrombotic microangiopathy, amyloidosis, immune-complex-mediated tubulointerstitial nephritis, ascending tubulointerstitial infection, opportunistic renal infection, and drug-induced nephrotoxicity.(31)

### Serum biomarkers

#### Autoantibodies

The main antinuclear antibodies related to SLE are anti-dsDNA antibodies, ribonucleic protein (anti-Smith or anti-Sm and anti-RNP) antibodies, and RNA polymerase antibodies.(21,27,29,30,32) Elevated ANA and anti-dsDNA antibody levels have been incorporated in the diagnostic criteria set out by the SLICC (Table 1). Other immunological criteria include: increased anti-Sm antibody levels; high antiphospholipid antibody levels (positive lupus anticoagulant test, false-positive rapid plasma reagin test, moderate to high anticardiolipin antibody levels, and positive anti-β2 glycoprotein I antibody testing); decreased complement levels (C3, C4, CH50); and positive direct Coombs test in the absence of hemolytic anemia.(29) Although autoantibodies are required in the diagnosis of SLE, their role in monitoring LN is unclear. Recent studies showed that LN may recur without prior increases in anti-dsDNA antibody levels.(21,32)

#### Complement system proteins

Complement system protein levels decrease in response to the activation of the classical complement pathway by IC deposited locally.(21,25,33) Decreased plasma levels of C3 and C4 have been traditionally associated with disease activity, particularly in proliferative LN.(21,32) However, these proteins are generally not very sensitive or specific to predict LN recurrence. Less than 25% of the children with low levels of C3 and C4 have recurring LN, and only 50% of the cases with recurring LN are preceded by drops in C3 and C4 levels.(21)

Increased circulating levels of erythrocyte-bound C4d (EC4d), reticulocyte-bound C4d (RC4d) or T cell-bound C4d are commonly seen in patients with active LN.(21,33) On the other hand, complement activation products such as C3a, C3d, and C5a were not as relevant as plasma C3 and C4 levels to clinical practice.(21) The decreased serum C1q levels seen in individuals with active LN may be associated with the presence of anti-C1q antibodies.(25,34) Patients cannot be diagnosed with LN based solely on anti-C1q antibodies.(35) However, when anti-C1q antibodies are associated with high levels of anti-dsDNA antibodies and low C3 and C4 levels in adults with SLE, the chances or renal involvement increase 15-fold.(36)

#### Creatinine

Serum creatinine is not particularly relevant in the diagnosis or assessment of LN.(21,32,36) However, progressive increases in serum creatinine have been associated with worse renal survival and must, therefore, be monitored in individuals with LN.(21,32,36)

#### Other serum markers

Interleukin (IL)-2, IL-6, IL-17, and IL-37 have been considered as potential biomarkers of LN.(37) However, further studies are required to determine the role of these markers in predicting the activity of LN or renal function decline.

### Urinary biomarkers

#### Urinary sediment (white and red blood cells)

Hematuria, red blood cell casts, and leukocyturia are generally suggestive of active glomerulonephritis in infection-free individuals.(21,29,30,32,38) The combination of hematuria and red blood cell casts is one of the diagnostic criteria for LN.(21,32,36) Recent studies indicated that glomerular hematuria may be associated with progression of renal disease.(39)

#### Proteinuria

Proteinuria is one of the diagnostic criteria for LN, although its absence does not rule out active LN.(21,32,36) Although lacking in specificity, urinary protein values above 1g/day may indicate severe renal involvement.(21,32,36,40) On the other hand, some studies suggested that significant drops in urinary protein after three or six months of therapy were associated with increased long-term renal survival.(21,32,38) Proteinuria has been related to inflammation, tubulointerstitial injury, and renal function decline.(21,32,36,41)

#### Other urinary markers

New urinary biomarkers such as soluble vascular cell adhesion molecule (sVCAM), angiostatin, ceruloplasmin, and osteopontin N-half (OPN N-half) were recently associated with LN activity.(37) When compared to the use of one single marker, combinations of some of these urinary biomarkers proved better in determining LN activity.(42-44) However, these biomarkers must be validated in longitudinal studies with greater numbers of patients, including children and adolescents.

#### Renal biopsy

Kidney histopathology is a valuable input in guiding treatment.(45) According to the recommendations published by the American College of Rheumatology (ACR) in 2012, renal biopsy must be ordered for patients with active SLE and/or suspected for renal involvement presenting proteinuria and/or hematuria or impaired renal function without an apparent cause.(5,30) In addition to these indications, renal biopsy may also be ordered for cases in which a diagnosis of LN has not been established due to inconclusive or dubious serological tests.(46) Kidney histopathology of individuals with LN shows glomerulonephritis associated with positive immunoglobulin tests for IgA, IgM, and IgG and complement system proteins C1q, C3, and C4.(25,30)

In some cases, particularly for pediatric patients with active renal injury, serial renal biopsies may be clinically relevant.(47) Renal biopsy helps monitor tissue alterations that may indicate changes in LN classification, disease activity, extent of irreversible chronic alterations, and progression of renal injury in response to immunosuppressant therapy.(21,32,36) Histopathology must include tests for immune deposits of IgA, IgM, and IgG, complement fractions C3, C1q, C4d, C5b9, and fibrinogen, in addition to electron microscope examination.(3,21,25,32,36,48)

## Pathology

**Figure 2:**
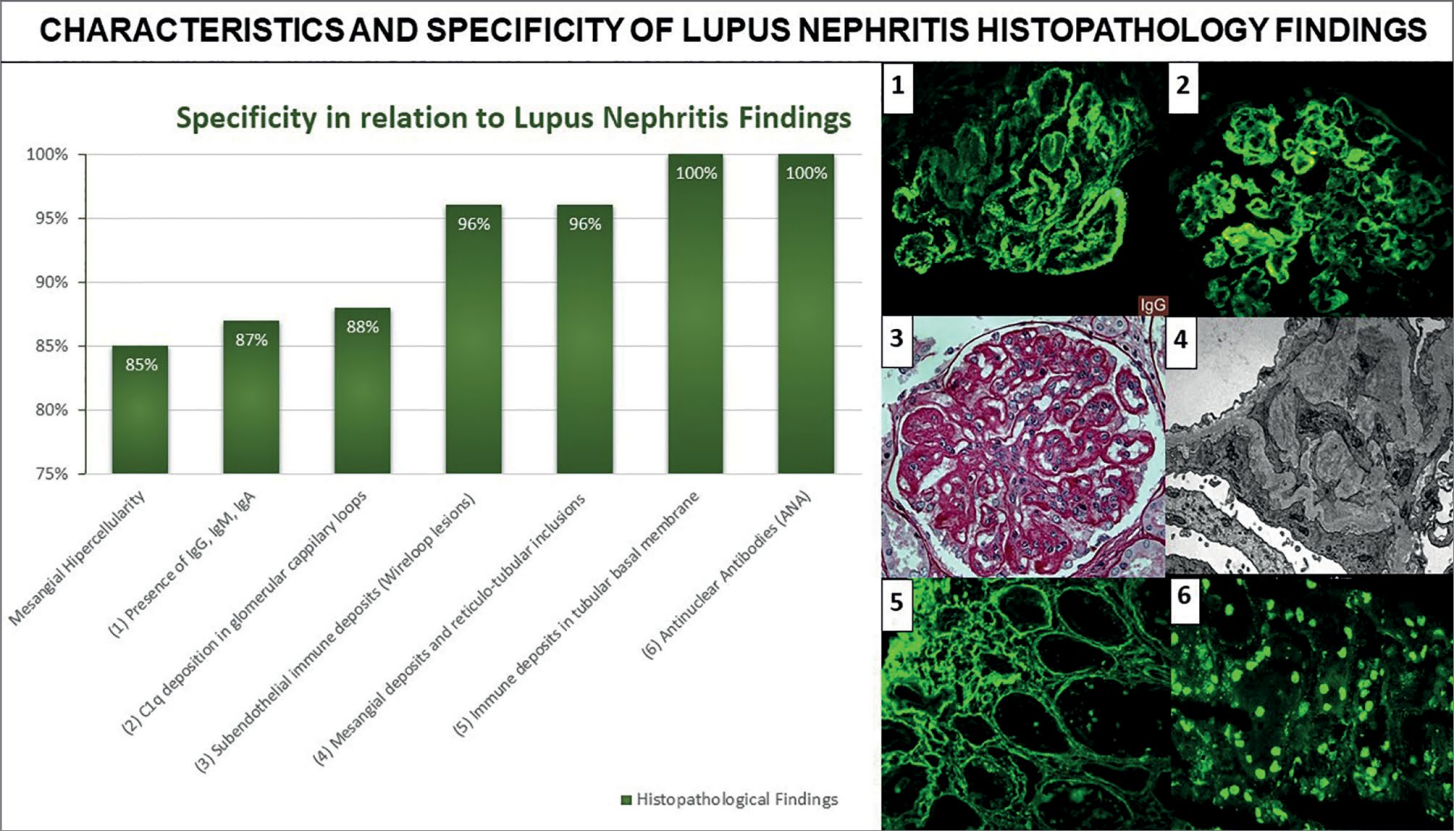
Characteristics and specificity of the histopathology of lupus nephritis. Adapted from Jennette et al. (1983).^68^

LN is characterized by the following features: systemic production of autoantibodies, complement disorders, circulating IC deposition, cell injury and podocyte, mesangial cell, endothelial cell, and tubulointerstitial component adaptive responses (Figure 2).(49-52)

### Morphological classification

**Figure 3:**
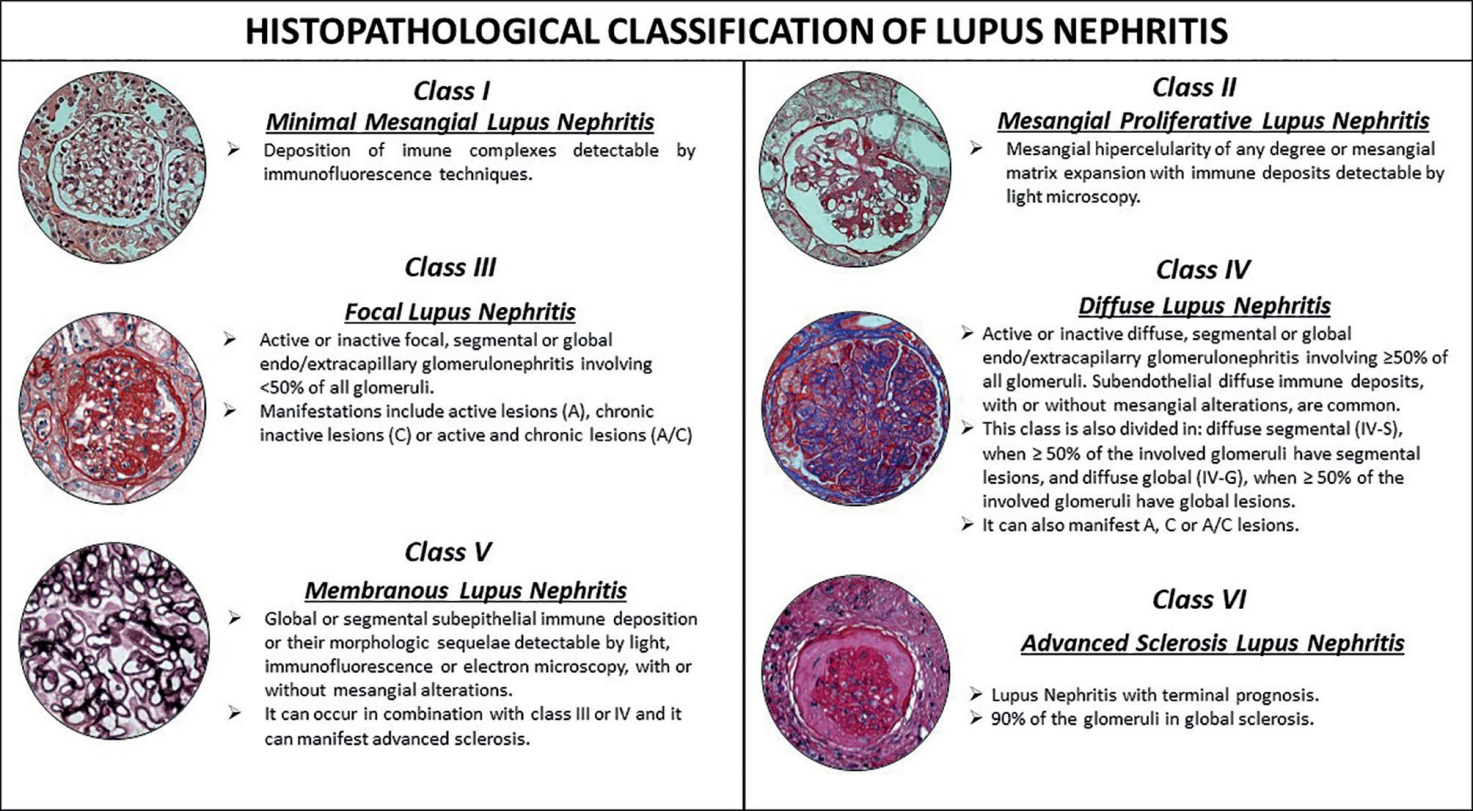
Histopathology classification of lupus nephritis according to the criteria established by the International Society of Nephrology and the Renal Pathology Society (ISN/RPS) in 2003 revised in 2018^48^,^49^,^51^.

The recommendations of the International Society of Nephrology (ISN) and the Renal Pathology Society (RPS) designed in 2003 and revised in 2018 (Figure 3) are currently used as the basis for the classification of LN.(49,50,51) The recently reviewed classification for LN introduced changes to the indicators of disease activity and chronicity, as shown in Table 2.(51) Some studies have advocated the inclusion of other classes and the incorporation of descriptors related to prognosis of therapeutic response, such as thrombotic microangiopathy, lupus podocytopathy, crescentic disease with or without antineutrophil cytoplasmic antibodies (ANCA), details on deposition of complement factors, presence of membrane attack complex, and degree of tubulointerstitial injury.(23,48,52)

**Table 2:** Modifications proposed by the National Institutes of Health (NIH) to the system used to score lupus nephritis activity and chronicity

Activity Index	Definition	Score
Endocapillary hypercellularity	Endocapillary hypercellularity in < 25% (1+), 25-50% (2+), or > 50% (3+) of the glomeruli	0-3
Neutrophils/karyorrhexis	Neutrophils and/or karyorrhexis in < 25% (1+), 25-50% (2+), or > 50% (3+) of the glomeruli	0-3
Fibrinoid necrosis	Fibrinoid necrosis in < 25% (1+), 25-50% (2+), or > 50% (3+) of the glomeruli	(0-3)x2
Hyaline deposits	Wire loop lesions and/or hyaline thrombi in < 25% (1+), 25-50% (2+), or > 50% (3+) of the glomeruli	(0-3)x2
Cellular/fibrocellular crescents	Cellular and/or fibrocellular crescents in < 25% (1+), 25-50% (2+), or > 50% (3+) of the glomeruli	0-3
Interstitial inflammation	Interstitial leukocytes in < 25% (1+), 25-50% (2+), ou > 50% (3+) of the cortex	0-3
Total		0-24
Chronicity Index		0-3
Glomerulosclerosis score	Global and/or segmental sclerosis in < 25% (1+), 25-50% (2+), or > 50% (3+) of the glomeruli	0-3
Fibrous crescents	Fibrous crescents in < 25% (1+), 25-50% (2+), or > 50% (3+) of the glomeruli	0-3
Tubular atrophy	Tubular atrophy in < 25% (1+), 25-50% (2+), or > 50% (3+) of the cortical tubules	0-3
Interstitial fibrosis	Interstitial fibrosis in < 25% (1+), 25-50% (2+), or > 50% (3+) of the cortex	0-3
Total		0-12

Source: Adapted from Bajema et al., 2018.^51^

#### Class I(minimal mesangial LN) and Class II (mesangial proliferative LN)

LN classes I and II start from the formation of immune complexes such as circulating autoantibodies and/or self-antigens in mesangial cells.(23-25,48-51) The formation of mesangial IC may activate the classical complement pathway with the deposition of IC fractions, leading to variable degrees of mesangial cell and mesangial matrix proliferation.(23-25,48-51) Given the high regenerative capacity of mesangial cells, mesangial expansion does not progress and usually does not cause proliferative or sclerosing glomerular injury.(24) According to the ISN/RPS classification (2018), disease class I includes early glomerular involvement with minimal mesangial tissue injury mediated by IC.(51) In LN class II, injury mediated by IC is accompanied by hypercellularity and mesangial expansion.(49,50,51) These classes are associated with good prognosis. Treatment with immunosuppressants is generally recommended to manage extrarenal manifestations.(48) However, they may indicate the onset of progressive early stage injury, which warrants additional renal biopsies as proteinuria increases or as the glomerular filtration rate (GFR) decreases.(48,51)

#### Class III (focal proliferative LN) and Class IV (diffuse proliferative LN)

Proliferative LN (classes III and IV) is caused by the deposition of IC in the subendothelial space of the glomerular capillaries, either alone or in combination with the deposition of IC in the mesangial region.(23-25,48-51) Subendothelial deposition triggers the production of IFN-gamma by endothelial cells and, consequently, local inflammation and endocapillary hypercellularity.(51) Reticular aggregates - ultrastructural findings characteristically seen in scenarios of elevated IFN-gamma secretion - may also form.(53) Severe modes of the disease have been associated with crescentic formations stemmed from the rupture of glomerular capillary loops and leakage of mitogenic proteins (mainly fibrinogen) into the urinary space, with subsequent involvement of the parietal epithelium. Proliferative LN presents lesions that characterize activity and chronicity.(24,48-51) According to the ISN/RPS classification (2018), the criteria for activity are: endocapillary hypercellularity; glomerular neutrophils/karyorrhexis; fibrinoid necrosis; wire loop lesions and/or hyaline thrombi in the glomeruli; cellular and/or fibrocellular crescents; and interstitial inflammation.(51) The criteria for chronicity include: total score of segmental or global glomerulosclerosis; fibrous crescents; tubular atrophy and interstitial fibrosis (Table 2).(51)

Involvement with active (A) and/or chronic (C) lesions in less than 50% of the glomeruli is seen in LN class III.(24,48-51) Involvement of more than 50% of the glomeruli indicates LN class IV, which is subdivided into "S" - segmental glomerular injury, i.e., injuries affecting less than half of the glomerular tufts - and "G" - global glomerular injury, i.e., injuries affecting more than half of the glomerular tufts.(24,30,48-51)

Although other injuries may occur with LN, they are not used for classification purposes. Nevertheless, they may affect the choice of treatment.

- **Tubulointerstitial injury:** clonal expansion of B cells and plasma cells may trigger local production of antibodies and consequent increases in inflammatory response and formation of **tertiary lymphoid tissue**.(24,48-51) Deposition of IC along the tubular basement membrane also occurs. These injuries may help identify patients responsive to therapies targeting B cells, such as treatment with rituximab.
- **Vascular injuries** are common and may affect patient prognosis.(24,48-51,54,55) They originate from the deposition of IC in vascular smooth muscle cells and endothelial cells or by local complement activation. Five types of vascular injuries are often observed: **vascular IC deposits, arterionephrosclerosis, thrombotic microangiopathy, noninflammatory necrotizing vasculopathy**, and vasculitis. Other possible events include endothelial edema, transmural vasculitis with fibrinoid necrosis, mesangiolysis or fibrin thrombi and, enlargement of the lamina rara interna of the glomerular basement membrane seen with the aid of electron microscopy.(56) Some of these injuries may be related to manifestations of LN, including systemic hypertension, dyslipidemia, and thromboembolism.(24,30,48-51) Vascular injuries may help identify patients potentially responsive to eculizumab and thrombomodulin.(57)
- **Podocyte injuries** are common and stem from the loss of expression of the proteins present in the slit diaphragm (nephrin and podocin) and the disorganization of the podocyte cytoskeleton, culminating with the flattening, effacement, and microvillus transformation of the foot processes.(58) These changes can be viewed only through an electron microscope.(58) Affected patients develop marked proteinuria. Podocyte injuries may be used to identify patients potentially responsive to calcineurin inhibitors.
- **Crescentic injuries** arise from immune deposits or direct attack by inflammatory cells.(59) Between 30-100% of the patients with diffuse crescentic injury are positive for **ANCA and/or anti-myeloperoxidase antibodies**, showing overlapping SLE and ANCA-positive vasculitis.(60,61) This group of injuries may help identify patients potentially responsive to plasmapheresis and monoclonal anti-C5aR antibody.

#### Class V (membranous LN)

LN class V originates from the subepithelial IC deposition of either immune complexes transiting through the glomerular basement membrane or immune complexes formed locally to deal with podocyte antigens.(23-25,51) The complement system is then activated locally, usually with the formation of membrane attack complex (C5b-9), thickening of the glomerular basement membrane, and destabilization of the podocyte cytoskeleton.(25) LN class V is often associated with nephrotic-range proteinuria with or without hematuria. This class of the disease may occur in association with proliferative LN (Class III or IV).

#### Class VI (advanced sclerosing LN)

LN class VI results from the progression of lupus nephritis.(24) In this disease class, glomerular, vascular, and tubulointerstitial injuries from glomerulosclerosis are seen in more than 90% of the analyzed glomeruli.(24,48-51)

### Treatment

The therapeutic regimens tested for adults with SLE, although broadly recommended for juvenile SLE, may not be enough to manage the disease in pediatric patients. However, recent guidelines for the treatment of LN in children and adolescents are broadly based on consensus documents developed for the adult population.(4,18,27,38,40,62,63) The goals of LN therapy are: produce complete remission from the disease; produce maximal decreases in disease activity; minimize drug toxicity; prevent recurrences; prevent chronic kidney impairment; improve patient quality-of-life; and provide advice to patients and family members on the disease.(40,63) Complete remission is characterized by significant drops in proteinuria and improvement of the GFR after six to twelve months of treatment.(40,64) Partial remission is characterized by a reduction of 50% or greater in proteinuria and by the partial recovery of the GFR after six to twelve months of treatment.(40,64) Table 3 summarizes the therapeutic schemes for the different classes of the disease.(4,27,40,45,62,63)

**Table 3:** Summary list of treatment protocols for pediatric lupus nephritis according to histopathology classification^4^^,^^27^^,^^40^^,^^45^^,^^60^^-^62

TREATMENT SUMMARY	
LN Class I	A) Prednisone/prednisolone (< 0.5 mg/kg/day - no more than 30 mg/day). B) HCQ is generally not needed, but as other DMARDs, it is recommended based on the clinical manifestation of SLE.
LN Class II	A) Prednisone/prednisolone (0.25-0.5 mg/kg/day - no more than 30 mg/day), with gradual decrease.* B) HCQ (or another DMARD) is generally needed in case of persistent proteinuria, if there is no remission after three months of low-dose prednisone/prednisolone, or to manage extrarenal manifestations.
LN Classes III and IV, associated or not with LN Class V	Induction therapy: MMF or CP + corticosteroids Chemotherapy regimen (MMF or CP) - 3 options A) Euro-Lupus: intravenous CP (fixed 500 mg doses, every 15 days for three months - total dose of 3000 mg) followed by maintenance therapy with AZA. B) NIH: intravenous CP (500 mg/m^2^, increased to 750 mg/m^2^ if tolerated, every 30 days for six months - no more than 1 g) followed by trimestral administrations for another 18 months. C) SHARE: oral MMF (1200 mg/m^2^/day, adjusting dose to a maximum of 1800 mg/m^2^/day, for six months - no more than 3000 mg/day). Corticosteroid therapy - 2 options A) Intravenous methylprednisolone (30 mg/kg/dose for three consecutive days - maximum dose 1 g) followed by oral prednisolone/prednisone (0.5-1 mg/kg/day - no more than 40 mg/day, for four weeks) with gradual withdrawal.* B) High-dose oral prednisone/prednisolone (1-2 mg/kg/day - no more than 60 mg/day, for four weeks), with gradual withdrawal.* Maintenance therapy A) Oral AZA: doses of 2-3 mg/kg/day, no more than 150 mg/day. B) Oral MMF: doses of 500-3000 mg/day (teratogenic).
LN Class V	Induction therapy A) SHARE: oral MMF + prednisone/prednisolone in doses of 0.5 mg/kg/day, wth gradual withdrawal.* B) CP, CNI (cyclosporine /tacrolimus) or rituximab must be considered as options for non-responders. Maintenance therapy A) SHARE: oral MMF or oral AZA.
Nephroprotection	A) ACEi or ARBs to manage systemic hypertension and proteinuria.
Recurrence and refractory cases	LN Classes III or IV associated or not with LN Class V Mild surge A) Increase prednisone and consider changing DMARDs (HCQ, AZA, MTX). Severe surge A) Intravenous methylprednisolone. B) High-dose oral prednisolone/prednisone (1-2 mg/kg/day - no more than 60 mg/day), with gradual withdrawal after response.* Refractory disease A) Check compliance to treatment and keep current therapy in case of poor compliance. B) Replace therapeutic agent (MMF, intravenous CP or rituximab). C) Consider CNI (cyclosporine or tacrolimus) in selected cases.
Adjuvant therapy	A) Use sun screen daily; B) Routine lab workup for lupus activity; C) Periodic eye examination for patients on antimalarial medication; D) Daily exercises to help prevent cardiovascular disease; E) Balanced diet, rich in calcium and low in salt content; F) Supplementation with vitamin D, so that serum levels of 25-OH-vitamin D are above 30 ng/mL; G) Rigorous management of blood pressure and proteinuria with ACEi and/r ARBs when possible; H) Control dyslipidemia; I) Avoid nephrotoxic drugs (e.g.: non-steroid anti-inflammatory drugs - NSAIDs); J) Discuss reproductive health with the patient, including birth control, contraceptive medication, and sexually transmitted diseases; K) Consider administration of influenza, pneumococcal, and meningococcal vaccines; L) Assess changes in cognitive performance at school and at home.

Notes: * Gradual withdrawal of prednisone/prednisolone: gradual decreases of 10-20% from the initial dose every one or two weeks to attain doses of 5-10 mg/day after six months. AZA: azathioprine; ARBs: angiotensin II receptor blockers; CP: cyclophosphamide; CNI: calcineurin inhibitors; DMARDs: disease-modifying antirheumatic drugs; ACEi: angiotensin-converting-enzyme inhibitors; SLE: systemic lupus erythematosus; MMF: mycophenolate mofetil; LN: lupus nephritis; SHARE: Single Hub and Access point for paediatric Rheumatology in Europe.

Pediatric patients with SLE must be prescribed disease-modifying antirheumatic drugs (DMARDs) such as hydroxychloroquine (HCQ), methotrexate (MTX) or azathioprine (AZA).(4,18,27,38,40,62,63) HCQ is the most commonly prescribed drug for patients with juvenile SLE. The dosage for children is ≤ 5 mg/kg/day.(65) Children on HCQ must be examined regularly by an ophthalmologist.(4,63,64)

Renal biopsy is required in the development of LN therapy. However, extremely ill individuals cannot always undergo renal biopsies. Difficult-to-treat hypertension, massive proteinuria, and/or impaired function are indications of LN classes III and IV and, as such, must be treated even in cases where the patient cannot undergo a renal biopsy.(4,27,18,40,63)

#### Treatment of lupus nephritis classes I and II

The treatment of pediatric LN classes I and II consists of low-dose oral corticosteroids (prednisone/prednisolone < 0.5 mg/kg/day, no more than of 30 mg/day) for 3-6 months, followed by gradual withdrawal of medication.(4,27,40,63) HCQ is also recommended for patients with LN class II, while other DMARDs (MTX or AZA) should be considered in cases of severe extrarenal manifestations.(4,27,63,64) If proteinuria persists after three months of treatment, a new renal biopsy should be considered.(63) If LN progresses, some authors have suggested the use of mycophenolate mofetil (MMF), tacrolimus (TAC), and cyclophosphamide (CP).(4,27,63)

#### Treatment of lupus nephritis III and IV, associated or not with class V

LN classes III and IV are the most common and severe forms of LN in children and adolescents. The combination of proliferative LN and LN class V is highly prevalent in the pediatric population.(4,27,63) Since proliferative LN is usually linked to less-favorable prognoses, treatment strategies do not rely on the presence of an association with disease class V.(4,24,27) The treatment of proliferative LN is divided into two stages. The first stage includes **induction therapy**, with the purpose of attaining remission from the acute manifestations of LN.(4,27,40,63) The second stage is called **maintenance therapy**, whose purpose is to prevent recurrence and manage the disease in the long term.(4,27,40,63)

The main options for **induction therapy** are MMF and CP administered together with corticosteroids.(4,27,40,63) MMF and CP are equivalent in terms of efficacy and adverse events, although intravenous CP is more efficacious in the long term for children with severe SLE.(65) The long-term safety of intravenous CP in children is not entirely established. Gonadal toxicity by oral CP therapy is greater in sexually mature males and lesser in prepubertal children.(4,27,63) MMF is particularly useful when there is significant risk of gonadal toxicity.(64) Intravenous CP may be the first choice when there is risk of poor compliance to orally administered medication.(63)

There are two regimens for intravenous CP: the low-dose (intravenous pulses of 500 mg every 15 days, in a total of six pulses through a period of three months); and the high-dose protocol (intravenous pulses of 500-750 mg/m2/pulse; if 750 mg/m2/pulse is tolerated, a maximum dose of 1000-1200 mg/pulse may be attained, with a total of six monthly pulse injections).(63) The long-term outcomes of these regimens are comparable in terms of safety and efficacy.(66) The low-dose protocol may be preferred for Caucasian patients.(38,63) The SHARE group recommended that children and adolescents with proliferative LN be treated with oral MMF for six months (initial dose of 1200 mg/m2/day, no more than 2000 mg/day, increased to 1800 mg/m2/day, no more than 3000 mg/day, if response is not good).(63)

Regardless of the choice of CP or MMF, the induction scheme must be administered jointly with corticosteroids. The most commonly used corticosteroid protocols are: intravenous pulse of methylprednisolone (30 mg/kg/dose for three consecutive days, no more than de 1000 mg/dose), followed by oral prednisolone/prednisone (0.5-1.0 mg/kg/day); or high dosage oral prednisone/prednisolone (1-2 mg/kg/day, no more than 60 mg/day).(40,63) Although there is no difference in efficacy between intravenous methylprednisolone and oral prednisone/prednisolone, methylprednisolone should be preferred in more severe cases.(4,40,63) Oral corticosteroids must be kept for 3-4 weeks; good responders may be weaned from the medication in steps of 10-20% of the initial dose every one to two weeks, reaching doses of 5-10 mg/day after six months.(40,63)

The most indicated medication for **maintenance therapy** for proliferative LN are AZA (2-3 mg/kg/day orally, no more than 150 mg/day) or MMF (initial dose of 1200 mg/m2/day orally, no more than 2000 mg/day, increased to 1800 mg/m2/day, no more than 3000 mg/day, if response is not good), with similar efficacy and adverse effects observed in children and adolescents.(40,64) Some authors have indicated that MMF is superior to AZA in adults.(62,63,66,67) MMF has teratogenic effects, while AZA may be used during pregnancy. The ideal length of maintenance therapy is unknown. Consensus documents have indicated a minimum duration of three years.(63)

#### Treatment of lupus nephritis class V

The prognosis of membranous LN (class V) is better than that of proliferative LN.(24,28) There is no consensus around the treatment of LN class V in adults. Immunosuppression therapy with CP or MMF has been advocated, particularly for patients with nephrotic-range proteinuria.(40,63) Some patients may respond to monotherapy with corticosteroids.(4) The SHARE recommends oral MMF combined with low-dose oral prednisone/prednisolone as induction therapy for membranous LN in individuals with juvenile SLE, followed by maintenance therapy with MMF or AZA.(63) The long-term prognosis for patients with subnephrotic-range proteinuria and normal GFR is generally favorable, and treatment may be initiated with nephroprotective measures.(63)

#### Recurrent and refractory lupus nephritis

Therapy failure occurs mostly due to poor compliance to treatment.(40,46,63) Patient serum immunosuppressant level monitoring is recommended. Therapy changes may be introduced if the patient fails to respond after three months of treatment and poor compliance has been ruled out.(63) If the patient responds partially, wait for an additional 3-6 months for complete remission before changing the immunosuppressant regimen.(63) The reintroduction or increased doses of corticosteroids combined with DMARDs should be considered.(40,63) In cases of persistent, active or refractory proliferative LN - with or without membranous LN - MMF may be replaced with rituximab or intravenous CP; or intravenous CP may be replaced with MMF.(64) Although the efficacy of rituximab has not been confirmed in clinical trials, cohort studies with adults and children suggested the drug should be used in cases of refractory LN.(63)

#### Nephroprotection

Despite the lack of consensus on the matter concerning pediatric patients, prescription of angiotensin-converting-enzyme inhibitors and/or angiotensin II receptor blockers helps control proteinuria in adults with LN.(4,40,63)

## Conclusion

Although SLE is a rare disease in pediatric populations, its consequences may be severe and even fatal. Although the etiopathogenesis of LN in children and adults is similar, the disease is more severe in pediatric populations. Studies on LN affecting children and adolescents are required to detect new prognostic markers and define specific therapeutic schemes for individuals in this age range.
